# The Demands of Beauty: Editors’ Introduction

**DOI:** 10.1007/s10728-018-0360-3

**Published:** 2018-07-09

**Authors:** Heather Widdows, Fiona MacCallum

**Affiliations:** 1 Department of Psychology, University of Warwick, Coventry, CV4 7AL UK; 2 Department of Philosophy, University of Birmingham, Edgbaston, Birmingham, B15 2TT UK

**Keywords:** Beauty ideals, Beauty practices, Normalisation, Globalisation, Cosmetic surgery

## Abstract

This article introduces a Special Issue comprising four papers emerging from the Beauty Demands Network project, and maps key issues in the beauty debate. The introduction first discusses the purpose of the Network; to consider the changing demands of beauty across disciplines and beyond academia. It then summarises the findings of the Network workshops, emphasising the complex place of notions of normality, and the different meanings and functions attached to ‘normal’ in the beauty context. Concerns are raised here about the use of normal to justify and motivate engaging in beauty practices such as cosmetic surgery and ‘non-invasive’ procedures. Other workshop findings included the recognition of beauty as increasingly a global value rather than a culturally distinct ideal, and the understanding that there is no clear distinction between beauty practices that are considered standard and those that are considered extreme. These themes, especially the concerns around understanding of normal, are reflected in the recommendations made by the Network in its Briefing Paper, which are presented next in this introduction. A further theme picked up by these recommendations is the extent to which individuals who are not traditionally vulnerable may be so in the beauty context. Finally, the introduction highlights the key matters covered in the four papers of the Special Issue: regulatory concerns around cosmetic surgery tourism; the impact of digitally altered images from psychological and philosophical perspectives; the ethics of genetic selection for fair skin; and the attraction and beauty of the contemporary athletic body.

This volume is one of the outcomes of an AHRC Network Grant “The Changing Requirements of Beauty” which begin in September 2014. The network was led by four academics—one Philosopher (Heather Widdows), one Psychologist (Fiona MacCallum) and two lawyers (Melanie Latham and Jean McHale)—and was in partnership with the Nuffield Council on Bioethics. The Network continues, run by MacCallum and Widdows, and publishes a twice monthly blog with international reach and recognition [2]. The Network continues to enable collaboration between researchers across disciplinary lines and is becoming a first point of contact for those seeking expertise on issues of beauty. The papers in this Special Issue are part of the academic reflection and theorising which evolved from the Beauty Demands Network (the Network also published a “Briefing Paper” in June 2016, which was primarily aimed at policy makers and medical practitioners) [3]. While they stand alone as robust academic papers addressing particular topics, taken together as a body of work and alongside the Beauty Demands blog and briefing paper, they represent something far broader. They are part of a call to recognise the extent of the rising demands of beauty and just how transformative such demands are of individual’s life projects and plans, social expectations and structures, cultural values and assumptions, and of the institutional and policy frameworks which shape and frame what individuals and groups can be and do.

## The Beauty Demands Network

The purpose of the original AHRC grant was to bring together academics from across disciplines, with policy-makers, practitioners, activists and cultural actors to think collectively and creatively about the changing demands of beauty. There were a number of key questions which the network sought to address and hypotheses which it sought to test and explore. The starting presumption was that the demands of beauty were extending and becoming normalised, with little social or academic critique, and that while research had been carried out in various disciplines and worries expressed in a number of quarters (for instance, the Keogh Review’s worries about the rise in non-surgical techniques) there had been few attempts to address the phenomena across disciplines and to look collectively as well as individually [9]. The network positioned itself to explore these rising demands from this starting point as stated in the original proposal:

The assumption of the network is that beauty image is becoming ever more demanding and defining of women, and increasingly men, irrespective of their professions. The project will ask whether this is the case, and how this norm is constituted and how it impacts upon women. It will also ask whether the dominant beauty norm is increasingly a global beauty norm, and thus open to less cultural and sub-cultural resistance. The project is especially concerned with the role of technology in this. In particular, that procedures which were once regarded as ‘exceptional’ such as the use of surgery, are now regarded as ‘normal’ or even ‘required’ in certain contexts. Other increasingly demanding beauty requirements include hair removal and ‘non-invasive’ procedures to reduce the appearance of wrinkles. All of these procedures, whether ‘routine’ or ‘exceptional’, require time and effort to maintain, and arguably the ‘minimum’ required is increasing; fewer women go ‘bare faced’ or bare their flesh without hair removal. This project will explore the extent to which beauty norms are changing and how, as well as what this means for individuals, for regulation and for clinical practice. [1]

## Beauty Demands’ Assumptions, Hypotheses and Findings

To focus the deliberation of network members the project was organised around four key workshops.^1^ The intention was to ensure that key stakeholders engaged across disciplines and beyond academia in order to challenge local norms, whether disciplinary, institutional or social.

The first workshop, held at the University of Warwick, focused on body image and how our perception of bodies is changing. Participants included philosophers, psychologists, lawyers and cultural theorists, clinical psychologists, counsellors and artists. A key theme of this workshop was the rise of technological fixes to change our bodies, and the way that an image based culture is shaping our expectations. As communication and relationships in a visual and virtual culture evolve so our sense of self changes—with identity increasingly located in the visual self. Indeed, as one of us later argued, “in our private and virtual lives we must be camera ready so we can present our best self(ie)” [15, p. 59]. This workshop was an important scene setting event for the network. It provided a clear framework upon which the rest of the project built and which is still core to the Beauty Demands Network. Most importantly it embedded the commitment that cosmetic surgery should not be exceptionalised as a separate practice or set of practices, but that it should be conceptualised as a beauty practice on a spectrum with other beauty practices. The presented papers on tanning and skin-lightening served as reminders that many of the harms of beauty come from so-called routine practices. The impact of body image on our intimate relationships as well as our sense of self was brought home in discussions of the impact of unrealistic body expectations on sexual functioning and as reflected in the rise of revenge porn. Crucial too were the papers which showed the ubiquitous, continual and rising nature of the demands, and how normal and normalising these were—a theme which returned in every workshop and became a major finding of the Beauty Demands Network. An early draft of Herjeet Marway’s paper was presented at this workshop and the final version is the third paper in this volume [11].

The second workshop, held in London at the Nuffield Council on Bioethics, had a more medical focus. Participants included surgeons, nurses, general practitioners (GPs), and psychologists, as well as body activists and artists. Again a key and emerging theme of this workshop was the importance of ‘normal’, the very different ways in which it is used in different areas of the beauty debate, and why it is such a crucial concept. During the course of this workshop and in the virtual debates before and after, it gradually became clear that the term ‘normal’ had a pivotal role in beauty debates. Sometimes it was used overtly normatively—to justify what should be—at other times it was used as if it were a purely descriptive term, although reflection usually revealed that hidden normative assumptions were in play. For example, GPs and surgeons reported that ‘not being normal’ was a reason that women often gave for seeking cosmetic surgery. Indeed ‘not being normal’ was functioning in the discourse surrounding surgery as an acceptable *motivation* for seeking surgery and as a *justification* for why it should be performed (whether asserted by the doctor or the recipient), and as a *descriptive* benchmark.^2^

These three different uses of the term normal were deeply problematic and even contradictory. For instance, ‘normal’ used as a *descriptive* term functions to determine what surgery is justified. To use the term this way, as a gate-keeper for justified surgery, assumes some measure of objectivity or some shared consensus (at least amongst doctors making the decisions about the normality of a body part). Yet, while doctors do make judgements on whether a body part is abnormal (and abnormal enough that ‘correction’ is justified) there is in fact no standard for what might be normal for any given body part. For example, and in the context of the rise in requests for labiaplasty by young women, medical textbooks and guidelines fail to give a range for what is normal size for labia and tend to use idealised and stylised representations. Similarly, there are no objective criteria of what counts as normal, or average breasts, and indeed breasts come in very many shapes and sizes. In terms of breast functioning, size is immaterial for instance, when it comes to the likelihood of successful breast feeding. Accordingly normal is not about functioning; although there may be health reasons to reduce very large breasts which cause back pain and other physical problems. But in practice ‘normal’ refers *just* to the way breasts look. What looks ‘normal’ is not objective, but largely subjective. In a world where it is the breast with implants that we standardly see—in the media, in adverts and in celebrity social media—then it is likely that we are moving towards a notion of ‘normal’ not as ‘average’ taken across the range of actual breasts, but as ‘the breast we normally see’. This normalises, and even naturalises, the modified breast, such that gradually the unmodified breast is the one which looks abnormal.

Given that many cosmetic surgeons in practice regard ‘to be normal’ as an acceptable and ‘good’ reason for undergoing surgery, the normative nature of this discourse is significant. The extent to which this language has been learnt as a script to access cosmetic surgery merits further research. For instance, surgeons are keen to ensure they do not operate on patients with unrealistic expectations—indeed this is something which the General Medical Council (GMC) and Royal College of Surgeons (RCS) emphasise—but if the learned narrative is “to be normal”, simply asking patients why they want surgery will not reveal such expectations [5, 14]. Rather they will reply with what they know is acceptable, no matter what they actually feel. If ‘to be normal’ has become one of the acceptable narratives, regarded as an acceptable *motivating* and *justifying* reason for engagement in all sorts of beauty procedures, then ‘normal’ has lost its descriptive and demarcating function. ‘Normal’ has become nothing more than a value-judgement to signify what is desirable—used to access whatever breasts (bigger, smaller, perter, firmer) or labia (tighter, firmer, non-protruding, non-chaffing)—you wish. This is worrying as the current ethical assumptions which ground current practices wrongly assume that normal is a descriptive and realistic assessment. In addition, if normal increasingly functions in this way this may impact on those who fall outside the norm. It may produce an environment in which ever more is required to be ‘normal’, to be just good enough. This makes discrimination against those who cannot or will not conform to the demands of beauty more likely. There is no paper from this workshop in this special issue, but the worries about normalisation went on to become the basis of the core recommendations of the Briefing Paper [3].

The third workshop was held at the University of Birmingham, and addressed the ‘globalisation of beauty’. Participants at this workshop came from cultural studies, gender studies, law, psychology and sociology, and it was also attended by journalists. The workshop addressed the contentious issue of the extent to which beauty ideals are converging across cultures, whether they are Western or Global, and whether there are pockets of resistance. Not surprisingly there were divergent views on the extent to which the ideal was becoming global. However, there was broad agreement that beauty ideals are now negotiated in the context of globalisation and there is significant fluidity between and within communities; the notion that there are lots of culturally distinct and separate ideals was deemed unsustainable. Accordingly it is no longer plausible to argue that beauty ideals are mere taste, or to claim that changes to these ideals over time and place are sufficient to deny that there are emerging global trajectories. The rise in the demands of beauty—from daily practices such as skin-lightening and tanning—to more demanding practices such as surgery, is real, can be tracked, and needs serious academic attention. For example, while still a minority activity, the numbers of people ‘going under the knife’ (or needle) is increasing. Definitive data on recipient numbers is notoriously difficult to find, but all measurements suggest that the incidence is rising. The International Society of Aesthetic and Plastic Surgery (ISAPS) annually publishes estimated figures based on plastic surgeons’ survey responses. The total number of surgical and non-surgical procedures carried out in 2010 was estimated to be 14.1 million, rising to 23.6 million in 2016 [7, 8]. Of these, over half were non-surgical procedures (13.3 million); however, as most non-surgical procedures are not carried out by surgeons the total number of these (such as Botox and fillers) will be far higher. Not only are more people having such procedures, but perhaps more importantly more people wish to. One study of 5000 women found that over 45% would undergo cosmetic surgery if they could afford it [12]. So while there were diverse views about the extent to which global homogenization is happening, there was convergent recognition that beauty is an increasingly dominant global value, and all agreed that there was an exponential rise in the use of cosmetic procedures and beauty practices. The increasingly dominance of beauty as a core value is an issue which Andrew Edgar takes up in the final paper of this volume [4]. Clearly, the global nature of the beauty industry presents a challenge for regulation which is currently largely at national and regional level. Danielle Griffiths and Alex Mullock rise to this challenge in the opening paper of this volume [6]. First drafts of both these papers were presented at this workshop.

The final workshop of the network, held in Manchester at MMU, focused on the supposed distinction between routine daily beauty practices, and more extreme practices such as cosmetic surgery. Participants included philosophers, psychologists, sociologist, lawyers, and those working on health interventions. The purpose of this workshop was to explore the hypothesis that there is no clear distinction, nor way to distinguish between, practices which are daily and standard, and those which are often considered extreme. This work begun at this workshop, and presented by Widdows, has been completed in *Perfect Me: Beauty as an Ethical Ideal* [15]. In this volume we take up the related issues of how idealised images shape our perceptions of what is ‘normal’, the first draft of which was presented by MacCallum at this workshop [10].

## Beauty Demands’ Policy and Practice Recommendations

A key output of the Beauty Demands Network was the Briefing Paper (launched in June 2016 at the Nuffield Council on Bioethics), to policy-makers and practitioners [3]. The recommendations are divided into three sections; first, ethical issues, second, psychological issues and third, governance, regulation and legal issues. The summary of the recommendations are as follows:

- To recognise that ‘normal’ is a value judgement and not a neutral or descriptive term
- To improve understandings and representations of ‘normal bodies’
- To recognise that consent might be compromised by pressures to conform
- To recognise the potential for vulnerability in the beauty context
- To develop effective interventions that promote positive body images in school curriculums at all ages
- To develop media literacy in school curriculums and in the wider public
- To promote diversity of model and mannequin sizes and shapes
- To standardise training and qualifications required to administer so-called non-invasive procedures and cosmetic surgery
- To harmonise standards for premises in which procedures can be undertaken
- To set minimum standards for products which can be used in beauty practices
- To ensure that informed consent is sought personally by the practitioner carrying out the procedure; for all so-called non-invasive procedures as well as surgical procedures
- To require a ‘cooling off period’ for all procedures (cosmetic surgery and so-called non-invasive procedures)
- To separate roles of salespersons and advertisers from practitioners performing procedures
- To consider changing practice and policy with regard to advertisements to reduce risk of unrealistic expectations
- To put in place processes for better data collection, monitoring and reporting measures

Some of these recommendations, particularly those around consent, data, the need to regulate non-surgical as well as surgical procedures, and the need to ensure sales techniques are not coercive, are very familiar from the Keogh Review and the recent Nuffield Council Report [9, 13]. While policy needs to catch up with these reviews, there is now broad consensus that some action is needed. However, this is not true of all areas. For instance, there is no consensus with regard to whether regulation of advertising works, particularly in an on-line culture, or whether and what interventions in schools have lasting effects and do not compound the problem by highlighting the body further. The two recommendations which are unique to Beauty Demands and which have not been replicated elsewhere are those about the use of ‘normal’ and the extensive nature of ‘vulnerability’ in beauty.

The use, interpretation and perception of what is ‘normal’ was identified as a key ethical concern. As discussed above, the term ‘normal’ is currently used in ways that may appear to be neutral or objective, but are in fact underpinned by value judgments. In the full briefing document there are two recommendations around ‘normal’ and ‘normalness’:

Recommendation: Careful use of normal in all contexts.

Recommendation: Improving understandings of normalness (for instance, more literature and material featuring a wide range of breasts and/or other body parts in teaching and medical settings).

The second area where Beauty Demands’ recommendations differ from other reports is that we suggest that the extent of vulnerability when it comes to beauty is far more extensive than policy makes and practitioners have standardly recognised and that this has the potential to undermine informed consent. The recent Nuffield Council Report on Cosmetic Procedures calls for restricting access to all cosmetic procedures for the under 18’s [13]. However, this assumes, as much of health care policy and practice does, that those who are under 18 are uniquely and particularly vulnerable and in a way which adults are not. A number of papers presented in the Beauty Demands Network suggest that in fact significant vulnerability attaches to beauty at many stages of life. The situation in which people may find themselves can make them vulnerable, even if they might not normally be considered, or consider themselves, as such. When it comes to determining who is vulnerable in beauty contexts, it is likely that this does not map to standard medical assumptions. For instance, the psychological evidence shows that those who have low self-esteem and low body-satisfaction are likely to be more vulnerable (less resilient) to pressure to modify their bodies. Low body-satisfaction occurs throughout the lifespan and is often connected to, and triggered by, stress factors and life changes (such as divorce, unemployment or bereavement). While there are significant and particularly vulnerabilities which impact upon the young—they are far more likely to have embraced selfie culture and therefore suffer the added pressure of needing to succeed in the virtual world (to be ‘liked’)—they are also likely to conform more to the beauty ideal than older women. As wrinkles and other signs of aging become marks of shame, we might begin to see those who are older and who have more to do to attain the ideal start to suffer more. If this is so, current calls fail to address the real extent of vulnerability in beauty, and if vulnerability is a feature which can undermine the ability to consent, this is a direct challenge to the current reliance of informed consent as a way to ensure ethical practice in cosmetic surgery. The full recommendation in the briefing paper is:

Recommendation: Ensure practitioners are trained to recognise the potential for vulnerability, and to understand that vulnerability may well be found in those who would not usually be considered vulnerable.

## Beauty Demands’ Papers

The four papers in this workshop take forward questions raised in the Beauty Demands Network in academically innovative ways. Each is an example of how academic research can explore features of the emerging and increasingly dominant beauty ideal and ask about its consequences and implications for how we think about the body and the self and what we might need to do to respond to the rising demands of beauty.

The volume opens with a paper by Danielle Griffiths and Alex Mullock, “Cosmetic Surgery: Regulatory Challenges in a Global Beauty Market”, which addresses cosmetic surgery tourism. They focus on the increased numbers of UK individuals seeking cosmetic surgery abroad for predominantly financial reasons. Recipients travelling for surgery from the UK largely come from working class demographics. Griffiths and Mullock build on previous arguments that cosmetic surgery should not be regulated as if it were therapeutic surgery and contend that the “medical exception”, which exempts doctors from the scrutiny of the criminal law, should not apply. They argue that the risks of cosmetic surgery are magnified when travelling abroad for a number of reasons: it is more difficult to check the quality of the clinic and the qualifications of the surgeon; insurance and compensation may be more difficult to attain and pursue; informed consent may not be robust and may be compromised by package deals purchased in advance; and after-care will be limited after the recipient returns to the UK. Ultimately, Griffiths and Mullock recognise the limits of the law as a tool for addressing this phenomenon, particularly in the global context. They do not argue for banning this practice, or for excessively restrictive UK legislation as they recognise that little is to be gained by driving people to seek cosmetic surgery elsewhere. Yet they state:

But the legal is not redundant. Tighter regulation in the UK, including using the criminal law against surgeons who cause harm when they proceed with risky surgeries, will not prevent people from seeking services abroad. However, a domestic response would hopefully send out a symbolic message that such surgery is potentially dangerous and should therefore be treated with great caution.

In addition to making recommendations for a particular regulatory response, Griffiths and Mullock’s paper shows just how important cross-disciplinary discussions are when it comes to addressing the rising demands of beauty. Too often policy makers have regarded regulation as merely about managing the risks and harms to recipients. As a result policy-makers have largely ignored the social impact of engagement or the implications for those who do not engage. Griffiths and Mullock do not fall into this trap but recognise that “cosmetic surgery reinforces and heightens concern with body image and culturally prescribed standards of beauty, contributing to a youth culture that disdains aging and the elderly and upholds culturally specific standards of beauty”. Accordingly they emphasise the importance of regulation and the place which law—particularly symbolic law—has to play in such processes. They make a plea for recognising the importance of “indirect effects” “that would deter such surgery and thwart normalisation by delegitimising it. This would, we argue, make women think twice about seeking cosmetic surgery at home or abroad”.

The second paper in the volume is our own contribution, “Altered Images: Understanding the Influence of Unrealistic Images and Beauty Aspirations”. In this paper we consider the now frequent calls that altered images be labelled in some way. Almost every image we see, whether in print or virtually, is now modified in some way. Alterations range “from relatively minor retouching (whitening teeth and eyes, smoothing wrinkles, and erasing blemishes) to more dramatic modification (elongating limbs and slimming waists, thighs and arms)”. There have been numerous calls for regulation of such images and recent calls for the labelling of such images. The thinking behind this is that such labelling will alert consumers to the alteration, making them more aware of the artificial nature of the images and so less critical of their own body’s failure to live up to such unrealistic ideals. However, contrary to this hypothesis recent evidence suggests that labelling does nothing to reduce the negative impact on self-esteem which attaches to viewing idealised images and may even heighten negative effects. At first glance this appears counter intuitive: “Shouldn’t knowing images are altered reduce the expectation we place on ourselves to be better or perfect?”. Yet it is the case that “we continue to hold digitally modified images as ideals even when we are told they are not ‘real’”. In this paper we argue that this result is not as surprising, for psychological and philosophical reasons, as it might first appear.

The assumption that knowing images are unreal would lead us to discount them as relevant self-comparators turns out not to be the case. On the contrary, labelling results in us paying more attention rather than less to the features of the image which have been idealised. Accordingly “labelling images as digitally altered exacerbates negative social comparisons with ideal images, which in turn exacerbates criticism of our own appearance”. This psychological phenomenon fits with a philosophical account of beauty as an ethical ideal. If a woman has adopted beauty as her moral framework and judges herself morally with regard to how well she succeeds or fails in beauty then “knowing that beauty ideals are unreal and unattainable does nothing to reduce the wish to attain such ideals”. Recognising the place of images in creating the ideals which women strive for as symbols of the selves they want to be, rather than realistic images, is crucial to understanding the demands of beauty. As we put it in the paper:

It is this emotional commitment and investment in the ideal (manifested in the extent to which we judge ourselves and others by it) that helps to explain why the images which present us with instances of the perfect ideal do not lose their power simply because we know they are digitally retouched. Our imaginings of our perfect – or improved or better or good enough – self, the end point of the beauty ideal to which we are striving, has very little to do with what is actually achievable or likely to be achieved.

The third paper in the volume, “Should We Genetically Select for the Beauty Norm of Fair Skin?” is by Herjeet Marway. In this paper Marway combines a familiar topic from beauty discourse—that of the preference for ‘fair’ skin—with the bioethical discourse of genetic selection. She argues that while race is often recognised as significant, beauty is not, and by considering this ‘beauty preference’ through the lens of race the wrong of selection for fair skin is visible.

Marway focuses primarily on ‘black’ African–American women and ‘brown’ Indian women and argues that, despite prominent protest movements, fair skin remains a dominant beauty norm in these groups. She highlights the racial and colour hierarchies that underpin the connections between fairness and beauty and darkness and unattractiveness. She argues that “underwriting all this is a beauty norm for fair skin that exists in particular places and which arbitrarily values particular races or colours because of a history of racism and colourism—ultimately this is prejudicial”. Having documented the ubiquity of the practice of skin-lightening and the harms which attach to current practices, Marway turns to genetic testing and the possibility that individuals may use this technology to select embryos with fair skin. She draws parallels between cases of selection for disability and sex, and argues that in similar ways women may feel pressure to select against darker skin. Marway argues that selection for beauty reasons is no less trivial than selection for explicitly racial reasons. Her claim is that “selection for fair skin in the pursuit of beauty does include *some* ideas about what is better and what benefits might accrue to one’s child that are rooted in some of the problematic [racist] ways discussed”. She argues that selection for beauty cannot be separated from race as, “if the beauty norm for fair skin is selected for, in part, because of the discriminatory hierarchies and stereotypes associated with race or colour then such selection is not insignificant.” Accordingly Marway concludes that “selection for the beauty norm of fair skin is discriminatory and demanding” and “we should not make such selections in the pursuit of beauty”.

The final paper in this volume is Andrew Edgar’s paper, ‘The Athletic Body’. Edgar explores the ways that bodies—in this case the athletic body—carry the capacity for story-telling. In the paper Edgar compares the Olympian bodies of Johnny Weissmuller in the 1920s and Michael Phelps in the early 2000s. The differences between these bodies is striking, with Phelps’s characterised by a sculpted look of ‘clean bulk’, which exemplifies the requirements of firmness and smoothness of the contemporary beauty ideal. As Edgar notes, this body is not limited to sport but is “prevalent in cinema, fashion and, perhaps crucially, in the advertising of male health and fitness products”. This body—muscled, firm and buff—is a body which is presented as one to which men should aspire: “Clean bulk is the norm, against which others are judged, and towards which even non-athletes should aspire”. Edgar argues that this body is a normative body which is presented as “the personification of a meaningful life” and he suggests that it is no accident that this apes the superhero body. Edgar presents the athletic body as a mythical body (“the sporting monomyth”) which tells a particular story, of a contemporary hero, a story of performance and spectacle. In this fiction:

the professional athlete is an autonomous agent. The athlete has made a series of self-defining choices, successfully overcoming obstacles, and thereby realising a coherent and meaningful life. Like the superhero they have, ideally, an unerring awareness of the right thing to do…The hero wins. As such, their lives therefore come to exemplify a serious of good and meaningful choices.

He concludes that, while dominant, the story that this mythical body is invoked to tell, despite its claims to power, is in fact an impoverished account not only of the body but of the human condition. It is an illusion. It hides the reality of loss which shadows winning in sport, and is challenged by the female sporting body, in which power is transformed into a “sexualised spectacle”, and the illusion of autonomous power crumbles. In an increasingly visual and virtual culture, it is not just the aesthetic body which is inherently story-telling with regard to meaning and identity, but all bodies. Our body becomes the place where we tell our stories, we use it to tell others who we are and even what we are, “we write our*selves* – and our insecurities – on our bodies, for our bodies, actual, transforming and imagined, are ourselves” [15, p. 209].

## The future of Beauty Demands

The AHRC network grant which kicked off the Beauty Demands Network and from which this volume results continues to grow from strength to strength. That beauty matters, and matters more in our visual and virtual culture, is increasingly recognised. There is rising public concern about many aspects of the beauty ideal, not least the rise in the demand for cosmetic surgery, the routinisation, normalisation and naturalisation of non-surgical procedures, such as Botox and lip fillers, and the epidemic of body image anxiety which is debilitating and devastating. Beauty Demands provided an academic forum for debate beyond disciplinary silos, and while it may not have resulted in a single or consensus voice, it has provided an important intervention. Beauty Demands and its network members are increasingly speaking in public and policy fora, and the need for action, reflection and theorising in this space is every more pressing.

We are grateful to the AHRC for the initial funding and to all the network members and partners who continue to be active in driving Beauty Demands forward. We believe further research and engagement in this area is crucial and Beauty Demands will continue to be a place of discourse, debate and research for those working in this area, who bring different and divergent perspectives and views to the debate. We look forward to working with beauty scholars, practitioners, activists and policy-makers in the future and we hope you will enjoy this collection of papers.

## Footnotes

1 Full details of the workshops can be found on the Beauty Demands website (https://www.birmingham.ac.uk/generic/beauty/events/index.aspx).

2 ‘Recipient’ was the term the Beauty Demands Network used most often for those who access cosmetic procedures, in preference to ‘patient’ (which assumes a direct parallel with health-contexts), ‘client’ (which implies simple consumption as if buying such procedures is equivalent to purchasing a product or commodity) or ‘user’ (which has uncomfortable connotations of addiction and is overtly value-laden). We felt recipient, on balance, was preferable as it recognises there is a service being received, but is not passive, as patient is. However, the terminology remains problematic, and not all papers in the volume use this terminology.
